# Expression of Concern: Effects of Capsular Polysaccharide amount on Pneumococcal-Host interactions

**DOI:** 10.1371/journal.ppat.1013953

**Published:** 2026-02-17

**Authors:** The PLOS Pathogens Editors

Contrary to the declaration in the Data Availability statement, the data set provided on the Dryad page linked in this article [1] only includes a README file and does not provide the original raw data files supporting the article’s results. The corresponding author stated that the underlying data files were submitted to Dryad but that these files were subsequently deleted by the platform due to the submission process being incomplete, and stated that they no longer have access to the underlying data.

In the absence of the original raw data files supporting the article’s results, this article [1] does not comply with the journal’s Data Availability policy in place at the time this article was submitted. Therefore, the *PLOS Pathogens* Editors issue this Expression of Concern.
