# Correction: Effects of Capsular Polysaccharide amount on Pneumococcal-Host interactions

**DOI:** 10.1371/journal.ppat.1014141

**Published:** 2026-04-16

**Authors:** The PLOS Pathogens Editors

Following publication of the Expression of Concern on this article [1,2], author HE provided an updated Figshare link for access to the original data files supporting this article’s [1] results.

The updated Data Availability statement reads:

The link to our data is: https://doi.org/10.6084/m9.figshare.31558558. Nucleotide sequencing data has been uploaded to NCBI under BioProject PRJNA930766.

With this Correction, the *PLOS Pathogens* Editors consider the concerns underlying the Expression of Concern [2] resolved.

## References

[1] Zhu J, Abruzzo AR, Wu C, Bee GCW, Pironti A, Putzel G, et al. Effects of Capsular Polysaccharide amount on Pneumococcal-Host interactions. PLoS Pathog. 2023;19(8):e1011509. doi: 10.1371/journal.ppat.1011509 37540710 PMC10431664

[2] The *PLOS Pathogens* Editors. Expression of Concern: Effects of Capsular Polysaccharide amount on Pneumococcal-Host interactions. PLoS Pathog. 2026;22(2):e1013953. doi: 10.1371/journal.ppat.1013953 41701677 PMC12912550

