# Supplementary material for: Effects of Capsular Polysaccharide amount on Pneumococcal-Host interactions
Source: PLoS Pathog. 2023 Aug 4;19(8):e1011509. doi: 10.1371/journal.ppat.1011509 (PMC10431664; doi:10.1371/journal.ppat.1011509)
Supplement: Attachment — Submitted filename: Response to Reviewers_PPATHOGENS-D-23-00361_5_15.docx [file ppat.1011509.s001.docx]

Dear Editors:

Thank you for handling our manuscript "Effects of Capsular Polysaccharide Amount on Pneumococcal-Host Interactions". We also thank the reviewers for their thoughtful comments and suggestions, which were addressed in the attached resubmission (with changes highlighted) and point-by-point responses below.

Our analyses were based on mutants expressing *decreased* amounts of capsule. In the revised manuscript, we added new data (new Figure 7) in which we selected for a spontaneous mutant expressing *increased* amounts of CPS. This mutant showed enhanced colonization consistent with our other findings.

Other comments were dealt with by addition of new text.

**Editor’s Comments:**

Thank you very much for submitting your manuscript for consideration at PLOS Pathogens. As with all papers reviewed by the journal, your manuscript was reviewed by members of the editorial board and by several independent reviewers. The reviewers appreciated the attention to an important topic. Based on the reviews, we are likely to accept this manuscript for publication, providing that you address the review recommendations. For example, reviewer 3 suggested that insight into or comment on possible mechanism(s) by which capsule might promote colonization (beyond counteracting an IL-17-dependent mechanism) or bacteremia (beyond counteracting complement; e.g., see Kupffer cell interaction, PMID 35258522) would strengthen this interesting manuscript. Please also note that since the submission of this manuscript, an analysis of different capsular serotypes has been published, PMID 36943879, that may be appropriate to include in your comments.

We added reference to the new manuscript PMID 36943879. This study used primary human epithelial cells in culture. Like other *in vitro* studies, it shows (in Fig. 1F) that the expression of capsule inhibits adherence (labelled in this manuscript as colonization). Our findings *in vivo* show the opposite effect of capsule – it enhances colonization.

All other points are addressed below.

**Reviewer's Responses to Questions:**

**Part I - Summary**
Please use this section to discuss strengths/weaknesses of study, novelty/significance, general execution and scholarship.

Reviewer #1: This is a well written, concise manuscript examining the functional impact of variations in capsule thickness on steps in pathogenesis. The question is clearly posed in the context of opposing benefits in sites such as nasopharynx and blood. More capsule is believed to decrease interactions with host cells which impairs colonization and invasion but at the same time aid colonization and invasion by preventing phagocytosis, complement deposition and mucous clearance. This study has answers to this conundrum and indicates that capsule amount has a clear cutoff of benefit in each of several steps in pathogenesis.

The shielding assay is an excellent readout for capsule function.
Each assay is clearly explained and graphs show distinct differences.
The final graphs provide a nice peak into the clinical realm for relevance.

Reviewer #2: Streptococcus pneumoniae, part of the normal human nasopharyngeal microflora, is also a major pathogen whose capacity to cause invasive disease is critically dependent on production of a polysaccharide capsule, of which multiple serotypes exist. Invasive disease is invariably preceded by colonization of the upper respiratory tract, and such colonization also acts as a reservoir for transmission to new hosts. The critical role of the capsule in invasive disease is well understood, but its contribution to colonization and transmission has received only minimal attention to date. These early events are of fundamental importance to the host-pathogen interaction, particularly given that progression to invasive disease is an evolutionary dead end. The total amount of capsule presented on the pneumococcal surface impacts the degree of exposure of other surface components important for colonisation (e.g. adhesins), and the pneumococcus is known to be capable of regulating capsular expression by a variety of mechanisms. The generally held view has been that capsule might be less important in the nasopharynx than in the blood, where the invading organism is subject to highly efficient reticuloendothelial clearance mechanisms.
The present study turns this assumption on its head, providing convincing evidence that capacity to both colonise the nasopharynx in infant mice and to transmit to new hosts depends on maximal capsule expression. The authors use mutants in 2 different serotype backgrounds (4 and 6A) with reduced cps expression (due to point mutations in cpsE) and a genetically repaired fully encapsulated strain, to demonstrate that strains with <50% of the wild type level of cps expression exhibit poorer bacterial retention in the nasopharynx as well as more efficient clearance later in colonisation. This correlated with reduced transmission to new hosts. The strains with reduced cps expression exhibited greater accessibility of deeper cell wall structures to exogenous antibody and greater C3 deposition on the cell surface when exposed to normal mouse serum. Interestingly, differences in colonization were not seen in IL-17ra-/- mice, implying an important role for innate signalling in recruitment of neutrophils to facilitate bacterial clearance. The study provides important new insights into these critical events in the host-pathogen interaction, that are potentially applicable to a wider range of bacterial pathogens. The experiments are well planned and executed and the data are presented in a clear and logical fashion.

Reviewer #3: Strength
This paper describes the impact of pneumococcal capsule on colonization, transmission and invasive infection. The strengths include: 1) the importance of understanding the biology of capsule in pneumococcal carriage and invasive infection; 2) impact of capsule in shielding bacterial surface features; 3) using isogenic strains from two strain backgrounds for studying capsule.

Weakness
The paper is mostly descriptive in the nature of investigation from capsule amount, bacteremia in invasive infection, colonization, transmission and capsule thickness in wild strains. It lacks sufficient amount of data to explain how the amount of capsule makes those differences in various aspects of bacterial life/infection. In addition, the statement that the blood is the “dead end” for S. pneumoniae, and therefore thick capsule is meaningless for bacterial life and evolution seems to be too strong because bacteria in the blood may enhance bacterial transmission once being seeded into the lung environment from the circulation during bacteremic pneumonia.

We respectfully disagree. Pneumococcal pneumonia is generally lobar and a complication of aspiration of URT contents rather than bacteremia. Pneumococcal bacteremia is common infants (occult bacteremia) but this group seldom has accompanying pneumonia. In adults, bacteremia is a complication that follows pneumonia, not the other way around. Transmission is via contact with carriers in the URT, not LRT.

This entire submission shows how a thick capsule contributes to bacterial life and evolution (by promoting colonization and subsequent transmission).

Moreover, the authors mostly cited their own work but did not make necessary connection to the previous studies in capsule function (see below).

A number of additional references have been added (see below).

**Part II – Major Issues: Key Experiments Required for Acceptance**
Please use this section to detail the key new experiments or modifications of existing experiments that should be absolutely required to validate study conclusions.

Generally, there should be no more than 3 such required experiments or major modifications for a "Major Revision" recommendation. If more than 3 experiments are necessary to validate the study conclusions, then you are encouraged to recommend "Reject".

Reviewer #1: (No Response)

Reviewer #2: NIL

Reviewer #3: Major experiments
1. How does capsule thickness impact invasive infection? Can this be due to the impaired resistance of partial capsule to Kupffer cell capture in the liver? Bacterial burden in the blood, liver and spleen at various time points post ip inoculation should be very revealing.
The marked effect of capsule amount on invasive infection is shown in Fig. 2. Panels 2C,D show a clear dependence on serum complement *in vivo,* which correlates with complement deposition in Fig. 1E,F.

We did not assess how complement opsonized bacteria are ultimately cleared from the bloodstream, as the role of professional phagocytes and the reticuloendothelial system are well established. Since complement deposition is very rapid, we don’t see how looking at bacteremia at even earlier time points (<24 hrs) would add new insight.

We agree that for those pneumococcal types not cleared in a complement dependent manner, it would be interesting to look at the effect of capsule amount on interaction with Kupffer cells as described in PMID 35258552. This point and the reference were added to the discussion (lines 306-9). However, these types do not include types 4 and 6A analyzed in our study and would merit an entirely separate study with the few types (14 and 7F) shown to interact directly with Kupffer cells in a complement-independent manner.

2. Fig. 2B shows the importance of capsule in dealing with Th17-based immunity during colonization, how could this happen?
This was explained in the discussion (lines 295-300). “The effect of CPS amount on sustaining high colonization density and in prolonging colonization duration was shown here to be dependent on IL-17 signaling. This could be explained by IL-17-dependent recruitment of professional phagocytes(22). Thicker capsules could inhibit the non-opsonic uptake by immune cells by macrophage scavenger receptors such as MARCO during colonization(27).”

The identity of the IL-17-dependent cellular effectors of colonization clearance is unknown and is an active area of study in the lab.

3. Fig. 4 shows variable thickness of capsule in wild strains. Can these variations impact pneumococcal colonization, transmission and invasive infection?

Based on the results with up to 5-fold differences in capsule amount in isogenic strain, we speculate that the even larger variation among the clinical isolates would have major effects on colonization, transmission and invasive infection. It was not practical to rigorously test 30+ clinical isolates. Also, unrelated clinical isolates would be expected to have multiple differences besides capsule amount, making it difficult to draw any meaningful conclusions. That is why our main analyses were based on comparisons of isogenic strains.

To address this comment, we did additional experiments with one of the clinical isolates shown in Fig 6. (See lines 224-36 and new Fig. 7). A type 6A clinical isolate expressing a low amount of capsule was selected. “P592 was unable to cause bacteremia following IP inoculation at a low dose (10^2^ CFU) as expected (**Fig. 7A**). Following IP challenge with a high dose (10^7^ CFU), a variant P2797, with a colony phenotype of increased opacity, was obtained from blood culture of a single pup (**Table 1**). In contrast to its parent, P2797 was able to cause bacteremia following challenge at a low dose (10^2^ CFU). Immunoblotting showed that P2797 expressed ~11-fold more CPS relative to its parent (**Fig. 7B**). Whole genome sequencing and comparison to P592 revealed that these changes were associated with acquisition of a Thr321Ala missense mutation in CpsE of P2797. P592 colonized pups at relatively low levels (**Fig. 7C**). The selection for this mutation that increased CPS expression in P2797 allowed for more robust colonization. Together, these observations add to the evidence that capsule amounts permissive for invasive infection enhance colonization.”

**Part III – Minor Issues: Editorial and Data Presentation Modifications**
Please use this section for editorial suggestions as well as relatively minor modifications of existing data that would enhance clarity.

Reviewer #1: Suggestions:
In view of the emphasis on amount of capsule, one area would be beneficial to further discuss. The spontaneous mutants are depicted as having a set amount of capsule. Yet it is likely that they still undergo processes such as phase variation and capsule shedding. Is this the case or are these more like the phase locked mutants developed in this lab? Either way, do the authors think that a 40% decrease in capsule is in the same ballpark as the changes invoked by phase variation or shedding? Or are the physiologic processes much less impactful on capsule amounts overall and represent just fine tuning? This might be assessed using the shielding assay.
We added to the discussion (lines 322-4) that “Some of these differences could be due to opacity variation, which previously was reported to account for up to a 5.6-fold effect on capsule amount(18).”

We also added a statement to the Introduction about capsule shedding (lines 96-8). “There is also evidence that capsule expression is dynamic within the respiratory tract (19) and that it may shed in response to the epithelium(20).”

Note: It would be helpful to define CM in the first Figure legend.

Done

Reviewer #2: A minor irritation is the fact that the authors cite a couple of figure panels out of sequence (e.g. 1B cited after 1C, and 2E cited after figs 3 and 4). This may have been done to economise on the number of figures overall, but it doesn't help the flow of the paper. Perhaps the authors could consider whether some reorganisation (either text or figures) is in order at the revision stage.
We changed panel 2E into a separate figure (new Fig. 5) and refer to it in order as suggested. We kept panels Fig1 B,D in place to allow for ease of comparison to Fig. 1 panels A,C which use the same method with a different strain.

Table 2 and lines 189-192: The reduced transmission to new hosts in the cps mutant immediately raises the question as to whether this is a consequence of reduced shedding by the index pups, or reduced establishment of colonization by the contact pups, or both. If the authors have data on levels of shedding then it would be good to present it. The point is raised later in the Discussion (lines 266-269), but perhaps it could be addressed at the first opportunity.

We added to the discussion (lines 312-4) that “Shedding was not considered separately in this report, since colonization density was affected by capsule amount and is a main determinant of the number of shed *Spn*………Also, because of differences in colonization dynamics, we could not distinguish whether differences in transmission were due to altered shedding from the index pups or acquisition and retention by contact pups.”

It would also be helpful to have the time at which contacts were tested for colonisation to be specified in a footnote to Table 2, rather than having to refer to the Methods.
Added a footnote that ^#^Colonization was assessed 7 days (T6A strains) or 9 days (T4 strains) post-pneumococcal inoculation of index pups.
Line 474: there is a typo in the CFU/strain dose.

Corrected

Reviewer #3: Page 19 line 383, assessment of surface shielding with TEPC-15 should cite a reference or provide justification for the method.
This is a new method thoroughly described in this manuscript (lines 438-45)
Page 19 line 388, full spelling of PFA should be provided.
Corrected
Page 19 line 385, 391, “100uL” and “3uL” should be corrected with a space between number and unit; Greek letter should be used for micro.
Corrected
Some importance literature needs to be cited to connect this work to the rest of the world, such as Magee et al. (doi: 10.1128/IAI.69.6.3755-3761.2001) has described pneumococcal strain expressing ~20% of the WT capsule is sufficient for nasal colonization;

Thank you for pointing this out. We added to the Discussion (lines 263-70) that “In this regard, Magee and Yother previously examined the requirement for capsule during murine colonization and invasive infection(29). They concluded that isolates producing reduced levels of capsule remain capable of colonization and causing invasive infection. Their study, however, depended on type 3 strains, which are unusual among pneumococci because they produce highly mucoid colonies. While type 3 strains, which express very thick capsules compared to other types, retain fitness in vivo with ~20% of WT levels of CPS, the findings here suggest this is not generally the case.”

An et al. (doi: 10.1084/jem.20212032) report the importance of pneumococcal capsule in evading Kupffer cell capture in the liver in the context of invasive infection.
This reference was added (see above).
Figure 2, labeling of Y axis needs to be consistent across AB (bacterial burden) and CD (Bacteremia) if the two panels are talking about blood bacteria. Were blood bacteria in CD collected 24 hr post infection as in AB?
All panels now show Bacterial Burden in the Y-axis.
Being consistent with the expression of the same things across the paper, such as 100 CFU vs 102 CFU in Fig 2 and other figures.
Corrected for consistency
Many more typo errors in the text, figure legends and references should be carefully corrected.

The text has been reproofed

PLOS authors have the option to publish the peer review history of their article ([what does this mean?](https://urldefense.com/v3/__https:/journals.plos.org/plospathogens/s/editorial-and-peer-review-process*loc-peer-review-history__;Iw!!MXfaZl3l!ewgYV1uR12GC8cSHpY0I_xZOU7KgZ5P-9q09iyrOVcVz7l4XFJk-oUGJF7UN7HARJPGoQDZR6q1i7-DXozsdaA$)). If published, this will include your full peer review and any attached files.


If you choose “no”, your identity will remain anonymous but your review may still be made public.


**Do you want your identity to be public for this peer review?**

For information about this choice, including consent withdrawal, please see our [Privacy Policy](https://urldefense.com/v3/__https:/www.plos.org/privacy-policy__;!!MXfaZl3l!ewgYV1uR12GC8cSHpY0I_xZOU7KgZ5P-9q09iyrOVcVz7l4XFJk-oUGJF7UN7HARJPGoQDZR6q1i7-AUHMHNvA$).

Reviewer #1: **Yes:** Elaine Tuomanen

Thank you.

Reviewer #2: No

Reviewer #3: No
